# Assessment of Unconstrained Cerebrovascular Reactivity Marker for Large Age-Range fMRI Studies

**DOI:** 10.1371/journal.pone.0088751

**Published:** 2014-02-13

**Authors:** Sridhar S. Kannurpatti, Michael A. Motes, Bharat B. Biswal, Bart Rypma

**Affiliations:** 1 Department of Radiology, RUTGERS-New Jersey Medical School, Newark, New Jersey, United States of America; 2 School of Behavioral and Brain Sciences, University of Texas at Dallas, Dallas, Texas, United States of America; 3 Department of Biomedical Engineering, New Jersey Institute of Technology, Newark, New Jersey, United States of America; Hangzhou Normal University, China

## Abstract

Breath hold (BH), a commonly used task to measure cerebrovascular reactivity (CVR) in fMRI studies varies in outcome among individuals due to subject-physiology and/or BH-inspiration/expiration differences (i.e., performance). In prior age-related fMRI studies, smaller task-related BOLD response variability is observed among younger than older individuals. Also, a linear CVR versus task relationship exists in younger individuals which maybe useful to test the accuracy of CVR responses in older groups. Hence we hypothesized that subject-related physiological and/or BH differences, if present, may compromise CVR versus task linearity in older individuals. To test the hypothesis, empirical BH versus task relationships from motor and cognitive areas were obtained in younger (mean age = 26 years) and older (mean age = 58 years) human subjects. BH versus task linearity was observed only in the younger group, confirming our hypothesis. Further analysis indicated BH responses and its variability to be similar in both younger and older groups, suggesting that BH may not accurately represent CVR in a large age range. Using the resting state fluctuation of amplitude (RSFA) as an unconstrained alternative to BH, subject-wise correspondence between BH and RSFA was tested. Correlation between BH versus RSFA was significant within the motor but was not significant in the cognitive areas in the younger and was completely disrupted in both areas in the older subjects indicating that BH responses are constrained by subject-related physiology and/or performance-related differences. Contrasting BH to task, RSFA-task relationships were independent of age accompanied by age-related increases in CVR variability as measured by RSFA, not observed with BH. Together the results obtained indicate that RSFA accurately represents CVR in any age range avoiding multiple and yet unknown physiologic and task-related pitfalls of BH.

## Introduction

A broad definition of cerebrovascular reactivity (CVR) is the ability of cerebrovasculature to respond to chemical agents or CO_2_ changes in the form of vasodilation or vasoconstriction. While CBF is the physiological variable defining CVR, its correlate, the blood oxygen level dependent signal (BOLD) has an advantage of high signal to noise ratio for clinical applications [1], [2], [3], [4], [5]. BOLD is equally suitable for CVR measures since CMRO_2_ changes are negligible during most CVR challenges leading to a linear dependence between CBF and BOLD [6]. Earlier CVR assessments have been performed by injection of the vasodilating drug acetazolomide [7]. However, a feasible application of functional Magnetic Resonance Imaging (fMRI) to clinical assessment of CVR requires less invasive methods to evoke vascular activity. To this end, carbon-dioxide (CO_2_), supplied through a breathing-mask to subjects have been utilized. During scanning, CO_2_-air mixtures are introduced into the mask. Breathing air-gas mixture leads to perturbation of vasculature, hence avoiding intravenous drug injections [8], [9]. Gas breathing is less invasive, but patient-groups often do not tolerate the gas-mask and furthermore, CO_2_ perturbation takes a few minutes to produce significant BOLD response. Special groups such as chronic obstructive pulmonary disease (COPD) patients are often excluded from CO_2_ breathing studies using gas-masks thus narrowing the subject pool. Thus alternatives such as the breath hold (BH) task have been utilized to produce mild hypercapnia in a matter of seconds [10], comparable to breathing a mixture of CO_2_ gas and air and is generally considered a reliable CVR measurement method for fMRI applications [1], [2], [3], [11], [12]. But BH is still not an ideal CVR challenge as blood oxygen level dependent (BOLD) responses to BH differ between populations such as children and adults [13]. While the difference could mean potentially distinct CVR changes between children and adult groups, it has been difficult however to rule out alternative hypotheses. For instance, BH-induced responses vary depending upon the inhalation-depth prior to breath hold prompting parallel (external) measures of abdominal expansion quantifying breathing depth to verify BH performance [12]. In actual clinical applications that deal with inhomogeneous populations such as aged and in presence of disease, subject-related physiological differences such as temporal variations of oxygen and CO_2_ saturation in the alveoli and gas exchange between blood and alveoli during the BH episode remain difficult factors to account for. As lung volume contracts during BH, the physiological variables may vary in an unknown manner within large age-ranges. This is further exacerbated by the adoption of different styles of BH such as end-expiration or end-inspiration. Despite no major advantage of one BH style over another for fMRI studies [14], end-inspiration BH with short hold durations of 20 sec or below can be comfortably performed by younger and older populations without reaching the breakpoint. Overall, BH experiments and the variability of outcomes due to uncontrollable internal physiological factors must be critically considered and are often challenging to streamline, no matter how hard the subject or patient may try [15].

It is known from prior studies that subject-level CVR versus task responses have a linear relationship for CO_2_ [16] or BH [17] in younger individuals. Based on this empirical evidence, we hypothesized that BH-performance and BH-related physiological differences in subjects, if present may impact subject-level BH versus task linearity in an age-dependent manner. To test our hypothesis, fMRI-BOLD responses during motor and cognitive task performance were correlated with BH measures in the same cohort of younger (Mean age = 26 years) and older (Mean age = 58 years) subjects from our earlier study [18]. Distinct from earlier analysis at the voxel-level [18], subject-level correlations were performed in the present study after estimating the mean amplitude of fMRI-BOLD responses within the task-activated regions of interest in every subject. The results supported our hypothesis that subject-related physiology and/or BH-related performance differences impact subject-level CVR in an age-dependent manner since linear BH versus task relationships were observed only in the younger but not older individuals. To further ascertain the impact of BH- and/or subject-related physiological differences on CVR, an ‘unconstrained’ (task-free) marker namely the resting state fluctuation of amplitude (RSFA) [19], available from a rest fMRI scan, was used. RSFA, as a viable marker for CVR stems from multiple-independent studies on BOLD amplitude changes during CO_2_-hypercapnic challenge or BH indicating strong correlation with the resting state BOLD fluctuations and EtCO_2_ fluctuations in spontaneously breathing humans [2], [18], [19], [20]. The current study results indicated that subject-level RSFA versus task relationships were independent of age. Also, an increased CVR variability represented by RSFA was observed in the older individuals, which was not apparent during BH. These quantitative subject-level analyses indicate that BH may deviate from accurate CVR estimates in older individuals. We propose that the unconstrained CVR marker RSFA, available from a resting fMRI scan, well-tolerated by special populations, is a better option than BH for measuring CVR in larger age ranges.

## Materials and Methods

### Ethics statement

All experimental procedures were approved by the Institutional Review Board of the University of Texas at Dallas. Data from twenty four healthy subjects 12 younger (6 Male and 6 Female; mean age = 24years; range: 19–27 years) and 12 older (5 Male and 7 Female; mean age = 58 years; range = 55–71 years) with no history of head trauma and neurological diseases obtained as part of our earlier study [18] was used for the present study.

### Experimental Tasks

**Figure 1:**
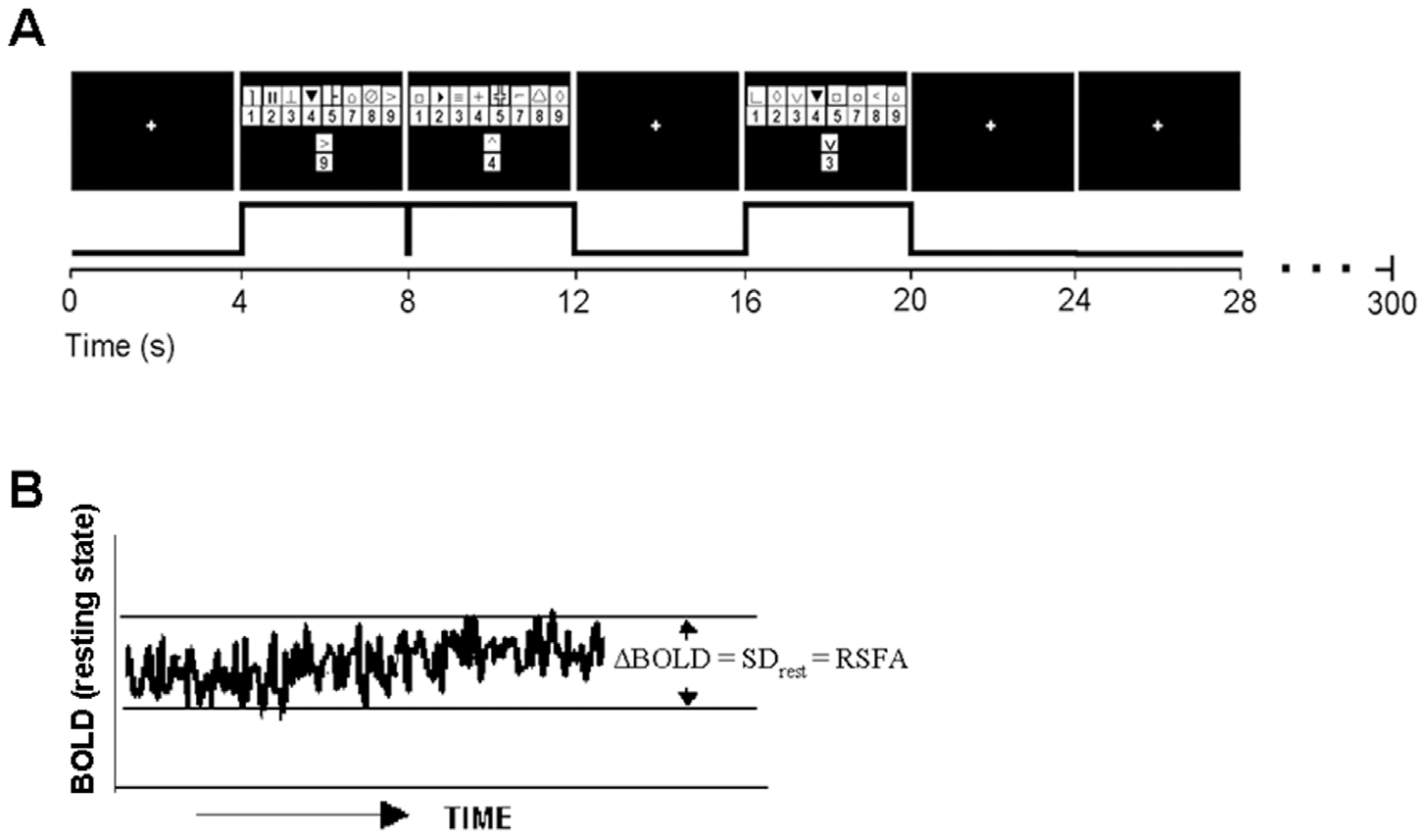
Schematic of the DSST task paradigm and RSFA estimates. **A.** During the DSST task, a digit-symbol key and digit-symbol probe appearing simultaneously for 4 seconds in each trial. The trials were jittered with 4-second periods of rest. **B.** Estimate of RSFA from the temporal standard deviation of the resting state BOLD time series.

Experimental tasks are explained in greater detail in our earlier study [18]. Briefly each subject performed a bilateral fingertap and a digit symbol substitution task (DSST) for the functional motor and cognitive tasks respectively. fMRI scans in the resting state and BH scans were also obtained in the same session for all subjects. The resting state scan was the first functional measurement, followed by the motor, BH and cognitive tasks. This order of scans was performed to avoid confounds that BH or cognitive tasks might introduce to the resting state response. Subjects remained relaxed with their eyes closed during the resting state scans. For the motor task, participants sequentially touched each finger of each hand to its respective thumb making one touch and release, at an easy rate of 0.5 sec in synchrony with the flashing circle. The experimenters observed the subjects prior to and during the scan where there was no group specific inability to maintain the minimal performance rate. The motor paradigm consisted of an initial 10 sec rest period followed by four repetitions of alternate epochs of 20 sec of bilateral fingertap and 20 sec of rest. BH consisted of a 40 second normal breathing period followed by three repetitions of alternate epochs of 20 seconds BH and 40 seconds normal breathing. Subjects performed an end-inspiration BH after inhaling a normal volume of air. Subjects were instructed not to inhale excessively prior to BH, but maintain their normal breathing volume [1], [21]. Using this style of end-inspiration BH, we have repeatedly demonstrated a non-biphasic response [1], [17], [21]. Normal inspiration significantly avoids the initial negative BOLD response typically observed in maximal-inspiration BH. An important reason we adopted end-inspiration BH is that it can be comfortably performed by younger and older subjects without reaching a breakpoint [15]. End-inspiration also minimizes reduction of lung volume and resulting alveolar contraction during the BH episode [22], [23]. This can be a significant confound as reduced lung volume reduces gas exchange between blood and the contracted alveoli during BH episodes. While the selective advantage of end-inspiration over end-expiration is still under debate, fMRI studies comparing the two observe significantly larger changes in ETCO_2_, BOLD and CBF responses during end-inspiration BH compared to end-expiration BH [14]. A modified Digit Symbol Substitution (DSST) paradigm consisting of a code table representing pairs of digits and nonsense symbols was used for the cognitive task [18], [24] (Figure 1A). In the DSST subjects were told that, if one of the digit-symbol pairs from the code table matched a probe-pair presented simultaneously beneath the table, they were to press a right-thumb button; otherwise, they were to press a left-thumb button. There were 52 trials in a single scanning run. On half the trials, the probe pair matched one of those in the code table and on the other half the probe-pair did not match one of those in the code table. The stimuli stayed on the screen for 4 sec followed by randomly varying inter-trial intervals of 0, 4, 8, or 12 sec. The cognitive task responses were well controlled. To wit, inter-subject variability of the DSST-induced BOLD responses within our study design has been observed to be relatively smaller than that during the fingertap motor task [25].

### Magnetic Resonance Image (MRI) acquisition

MRI measurements were performed on a 3T PHILIPS scanner. Gradient echo-EPI images were subsequently obtained during resting state, motor (fingertap), cognitive (DSST) and BH tasks. 32 slices were obtained in the axial plane covering the entire brain. Imaging parameters were: FOV = 220 mm, matrix size = 64×64, TR/TE = 2000/30 msec, and slice thickness = 4 mm. Number of acquisitions were motor task = 110, cognitive task = 150, BH = 120 and resting state = 110.

### Data Analysis

All analysis of the functional images were performed using AFNI [26]. Preprocessing and motion correction steps have been described in more detail in [18]. Briefly, a gamma-variate function was convolved with the task reference function and cross correlated with the BOLD signal on a voxel-wise basis to determine activation. During BH, the reference function was appropriately shifted to take into account the large hemodynamic delay during the BH response (Kannurpatti et al., 2002). Successful performance of the BH task by each subject was confirmed by a voxel-wise inspection of each subject data set during BH and observing the BOLD response to each epoch using the graph utility in AFNI. A threshold of p<0.01; Bonferroni corrected was considered for determining active voxels. This statistical threshold was optimal since it not only maximized the correlation between BH versus task and RSFA versus task responses with fewer outliers [17], but also preserved activity from tissue to a large extent. At this threshold, motor task-induced BOLD signal changes in the range 1–3% were the most prominent with 95% of activated voxels in the BOLD response range of <3%, confirming that the threshold was not biased towards large vessel signals [27]. Subject-level mean values were obtained after averaging across voxels within the task activated regions. Fractional changes in BOLD signal during task (% change) were estimated for the motor, cognitive and BH tasks which was different from the SD estimate obtained in our previous study [18]. The main reason for estimating the % change was to keep the analysis method consistent with other fMRI studies of CVR estimates. Fractional change in BOLD signal in response to task was estimated from the fit magnitudes of the general linear model (GLM). The temporal standard deviation (SD) was estimated from the resting state time series [18] that determined RSFA, (Figure 1B). All analyses were performed within the active voxels during the motor and cognitive tasks determined for every subject without any spatial normalization. For each subject, the frequency distribution of %BOLD change within the respective activated regions of interest during the motor and cognitive tasks was determined. Voxels making up the frequency count in frequency distribution analyses were obtained from the original functional images. Mean and SD of the frequency distributions were obtained for the younger and older subject groups. Coefficient of variation (CV = SD/mean) was estimated for various %BOLD changes within the motor and cognitive task activated regions. A similar procedure was carried out to obtain the distribution of RSFA within the activated regions. Equality of variance between the younger and older subject groups was tested using the Bartlett's Test with a p<0.05 required for a significant difference.

## Results

A subject-wise analysis was followed in this study as previous age-dependent BH-task relationships have shown no apparent differences at the voxel level [11], [18], [19], [28], [29]. Furthermore, different CVR variables (eg., CO_2_, resting state fluctuation of amplitude-RSFA, amplitude of low frequency fluctuations-ALFF, BH) that significantly correlate among themselves do not show age-dependent differences in correlation at the voxel-level [18], [19], [28], [29]. Hence variables such as the BH response which are subject- and task-dependent, are bound to be more sensitive to age-dependent differences at the subject level. Group activation maps for the current cohort of subjects across all the tasks and behavioral measures on the cognitive task have been published in our earlier studies [18], [30].

**Figure 2:**
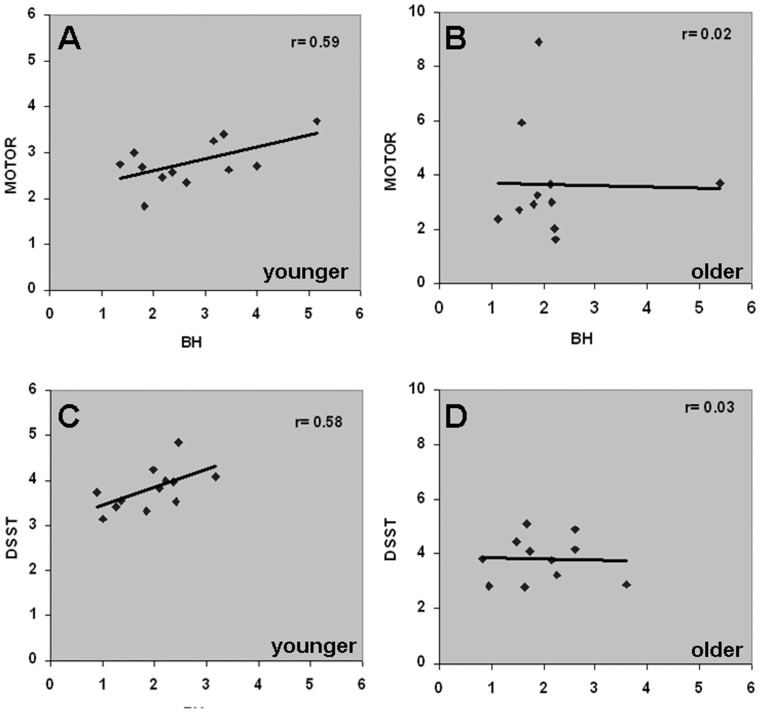
Relationship between the subject-average fractional BOLD signal change (%) during the motor (fingertap) and cognitive (DSST) tasks versus BH challenge. **A, C**. in younger subjects and **B, D**. in older subjects. A significant linear correlation was observed between the motor task versus BH (r = 0.59; p<0.05) and cognitive task versus BH (r = 0.58; p<0.05) in the younger subjects. However, no correlation was observed between the motor task versus BH (r = 0.02) and the cognitive task versus BH (r = 0.03) in the older subjects.

Significantly activated voxels in every subject during the motor and cognitive tasks were considered as regions of interest and the average motor and cognitive task-induced %BOLD signal change was determined. Similarly, average BH-induced %BOLD signal change was also determined from the respective motor and cognitive task activated regions from every subject. Figure 2 indicates the scatter plots of the mean BOLD response from the motor-task activated regions and the mean BH response from the same regions in every subject. From Figures 2A and 2C, a significant linear relationship was observed between BH versus fingertap (motor) task responses (r = 0.59; p<0.05) and BH versus DSST (cognitive) task responses (r = 0.58; p<0.05) respectively in the younger subjects. However, this relationship was disrupted in the older subjects (Figures 2B and 2D). To assess the influence of outliers on the results, we performed our analysis after removal of data points that deviated by more than 3 standard deviations from the group mean. This led to the identification of two subjects. Removal of the outliers (data point: 5.4, 3.7 and data point: 1.9, 8.9; Figure 2B), led to an improvement in the linear correlation from 0.02 to 0.2. However, despite removing outliers, the linear correlation was not significant. Similarly, removal of an outlier subject (data point: 3.6, 2.9; Figure 2D) led to an improvement in the linear correlation from 0.03 to 0.29. However, despite removing the outlier, the correlation was not significant. To test whether the lack of linearity in the older group was robust, we pooled all subjects into a single group and performed the linear regression. In this single large age-range group, we observed that the linear BH versus task relationship was still disrupted within the motor (Figure S1A) and cognitive task activated areas (Figure S1B).

**Figure 3:**
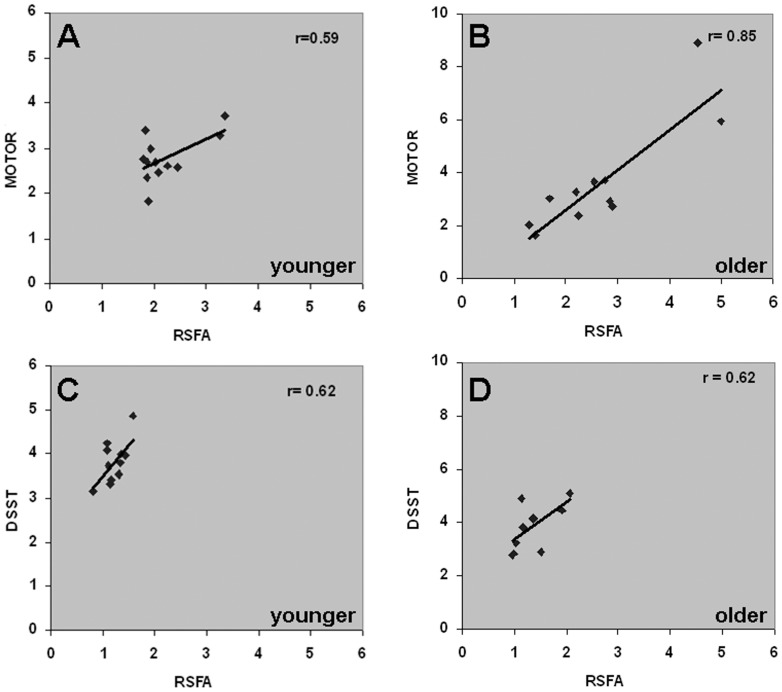
Relationship between the subject-average fractional BOLD signal change (%) during the motor (fingertap) and cognitive (DSST) tasks versus RSFA. **A, C.** in younger subjects and **B**, **D**. in older subjects. A significant linear correlation was observed in motor task versus RSFA (r = 0.59; p<0.05) and cognitive task versus RSFA (r = 0.62; p<0.05) in the younger subjects which was intact in the older subject's motor task versus RSFA (r = 0.85; p<0.05) and cognitive task versus RSFA (r = 0.62; p<0.05).

Using the resting-state scan from each subject, subject-wise mean RSFA was calculated from the voxel average of the motor and cognitive task-activated areas. Figures 3A and 3C show the scatter plots of the subject-wise RSFA versus the %BOLD response during task. A linear relationship was observed between RSFA versus motor task response (Figure 3A; r = 0.59; p<0.05) and RSFA versus cognitive task response (Figure 3C; r = 0.62; p<0.05) respectively in the younger subjects. A comparable linear relationship was observed in the older subjects within the motor (Figure 3B; r = 0.85; P<0.05) and cognitive task activated areas (Figure 3D; r = 0.62; p<0.05). In the larger age range, the linear RSFA versus task relationship was intact within the motor (Figure S1C) and cognitive task-activated areas (Figure S1D).

**Figure 4:**
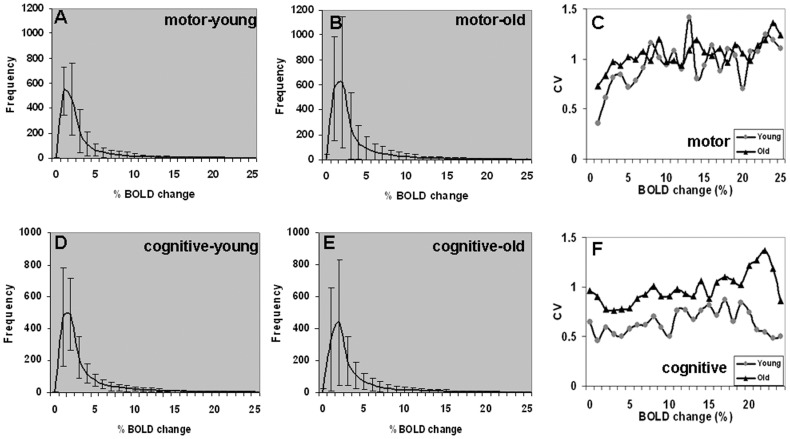
Distribution of the BH-induced BOLD response and its variability in the younger and older subjects within the motor and cognitive task activated areas. **A.** in younger-motor, **B.** in older-motor, **D.** in younger-cognitive, **E.** in older-cognitive. **C.** BH response variability within motor task activated area, and **F.** BH response variability within the cognitive task activated area. (coefficient of variation = CV = sd/mean).

Possible causes for the disruption of the linear relationship between BH versus motor and BH versus cognitive task responses in the older subjects were investigated based on the hypothesis of subject-specific physiology and/or BH-performance difference. The percent-wise distribution of the BH-induced BOLD responses within the motor task activated areas was estimated in the younger and older subjects (Figure 4A and 4B) along with a coefficient of variation (CV = sd/mean) estimate for different levels of BOLD amplitude changes (Figure 4C). A similar estimate of the %-wise distribution within the cognitive task areas was estimated for the younger and older subjects (Figure 4D and 4E) along with the CV estimate for different levels of BOLD amplitude changes (Figure 4F). No significant difference was observed in the BH-induced BOLD response variability within the motor task activated areas in the younger and older subjects (p<0.99, Bartlett's test of equality of variance). A similar result was observed for the BH outcome in the cognitive task activated areas (Figure 4D–F) where moderately larger BH-induced BOLD variability was observed in the older subjects, but the difference was not significant (Bartlett's test; p<0.7).

**Figure 5:**
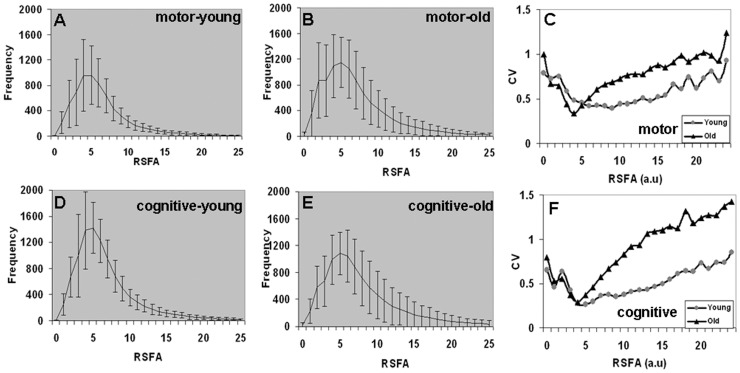
Distribution of RSFA and its variability in the younger and older subjects within the motor and cognitive task activated areas. **A**. in younger-motor, **B**. in older-motor, **D**. in younger-cognitive, **E**. in older-cognitive. **C**. RSFA variability within motor task activated area, and F. RSFA variability within the cognitive task activated area. (coefficient of variation = CV = sd/mean).

As RSFA is independent of task performance (unlike BH), we applied it as an ‘unconstrained’ (task-free) measure for a comparative analysis of RSFA variability within the motor task activated region. RSFA distributions within the motor task activated areas was estimated for the younger and older subjects (Figure 5A and 5B) along with coefficient of variation (CV = sd/mean) estimates (Figure 5C). Significantly greater BOLD variability was observed in the older than younger (Figure 5A-C) subjects (Bartlett's test, p<10^−3^). A similar trend in RSFA variability was observed in the cognitive-task activated areas (Figure 5D–F) with significantly larger RSFA variability in the older compared to younger subjects (Bartlett's test, p<0.05). Higher RSFA variability in the older was consistent with earlier studies indicating more variable hemodynamic response functions and resting state noise among older subjects compared to younger [31], [32]. The results from Figures 3 and 4 indicate a distinct RSFA variability compared to BH between the younger and older for both motor and cognitive areas. While RSFA variability was significantly different between the younger and older, BH did not show such a trend.

**Figure 6:**
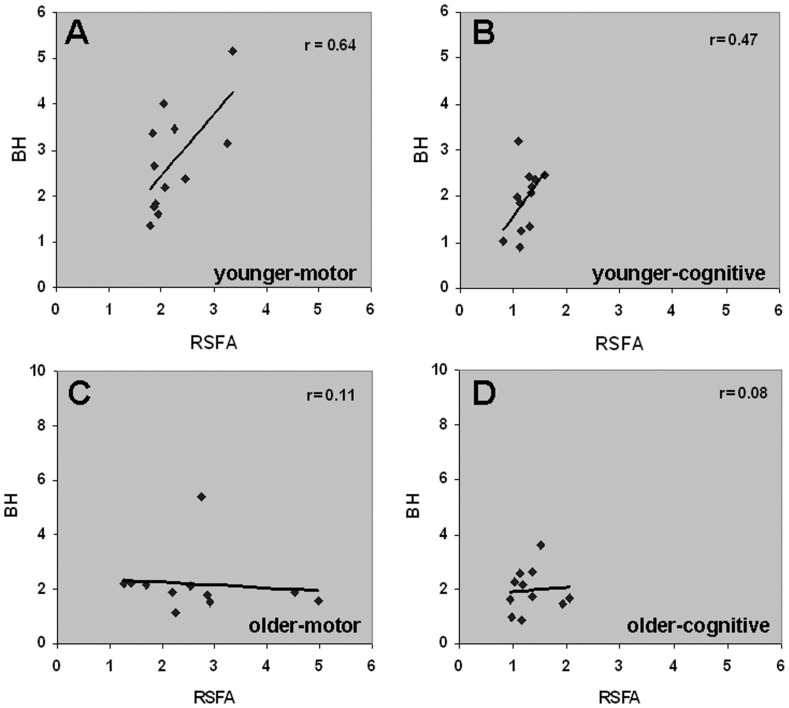
Correspondence between BH and unconstrained-RSFA representing CVR. A significant linear relationship between BH versus RSFA was observed within the motor and cognitive task activated areas in the **A**–**B.** younger subject group (r = 0.64; p<0.03; r = 0.47; p<0.12) which was disrupted in the **C**–**D.** older subject group (r = 0.11; r = 0.08).

Further comparisons of the subject-wise relationships between BH and RSFA were performed. As indicated by the results in Figure 6A, a significant linear relationship was observed between BH versus RSFA only in the younger subjects within the motor task activated areas. The linear relationship within the cognitive task activated areas was apparent but did not reach significance (Figure 6B). BH versus RSFA however did not show any linear relationship in both motor and cognitive task activated areas in the older group (Figure 6C–D). In the larger age range, no linear BH versus RSFA relationship was observed within the motor (Figure S2A) and cognitive task-activated areas (Figure S2B). Thus the lack of a linear relationship between BH versus motor and BH versus cognitive task responses in the older may be linked to subject-specific physiology and/or BH-task, which might not accurately reflect age-related CVR changes within specific brain structures.

**Figure 7:**
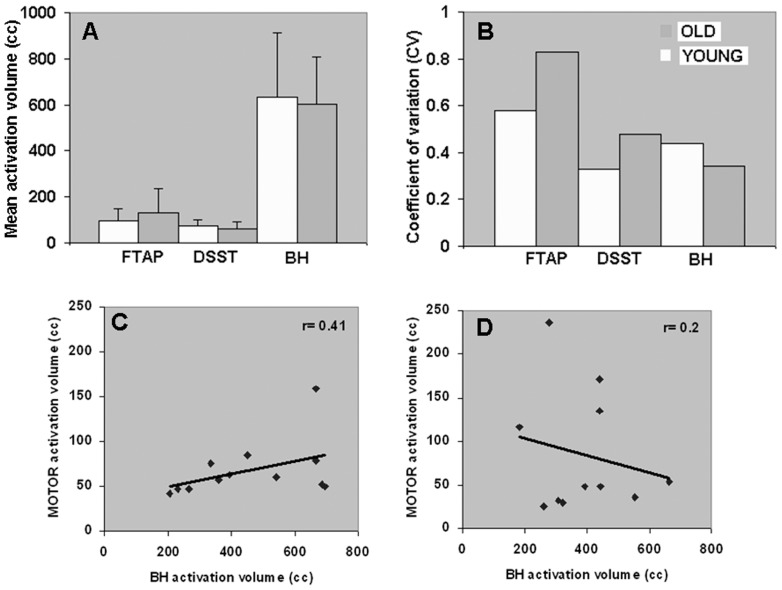
Activation volume and their relationships. **A.** Mean activation volumes did not differ significantly between the younger and older subjects during the motor, cognitive and BH tasks. **B.** no differences were observed in the variability of activation volumes except the motor task; motor (P<0.03), cognitive (P<0.72) and BH tasks (P<0.99); Bartlett's test of equality of variance. **C.** BH-motor task relationship defined by activation volumes in younger subjects and **D.** in older subjects. A significant linear correlation was observed in the younger group (r = 0.41; p<0.05) while no significant correlation in the older group (r = 0.20; p<0.1).

Since activation volumes represent statistical test parameters depending on both signal and noise levels, we compared the activation volumes during all tasks in both groups. No significant differences were observed in the mean motor, cognitive or BH-induced activation volumes between the younger and older groups (Figure 7A). Note that the present analysis determined the activation volume after statistical parametric mapping and established the number of activated voxels in every subject. This subject-wise determination of activation volume was different from our prior map-wise analysis of the same cohort of subjects wherein group activations determined after averaging the z-scores of all subjects led to a significant decrease in the motor and cognitive task-induced activation volumes in the older compared to the younger [18]. Except for the motor task, activation volume variability was not significantly different for all other task-related BOLD responses between the younger and older (Figure 7B; p<0.03; Bartlett's test). As the motor task showed significant variability between the groups and was self-paced (i.e., easily performed by both the younger and older subjects), the spatial extent of the motor task-activated BOLD responses would represent more of the vascular component of variability than the neural component based on our prior study using a block design paradigm [27]. BH versus motor task response relationships determined using the activation volume also showed a significant linear trend in the younger subjects (Figure 7C; r = 0.4; p<0.05) but not in the older subjects (Figure 7D; r = 0.2; p<0.1).

## Discussion

This study highlight a number of methodological and physiological issues related to the reliable estimation of CVR by BH in human populations of different ages and suggest an internal standard such as RSFA as an alternative.

### BH performance/physiological confounds in CVR estimation

BH has been frequently used in fMRI to test CVR [1], [2], [3], [11], [33]. However, when considering aged individuals or with disease, BH-induced BOLD responses may vary due to physiological factors such as differences in oxygen and CO_2_ gas exchange in addition to performance differences in the BH task itself [12]. In younger and more homogeneous groups of human subjects, CVR assessed as a change in BOLD or CBF per end-tidal CO_2_ change can be more accurate when using CO_2_ inhalation methods [34]. However, continuous end-tidal CO_2_ during BH durations are not available and need to be assumed as time-linear [35]. An important subject-related physiological factor is lung volume contraction during BH, impacting gas exchange between bood and alveoli [22], [23]. This is a difficult variable to account for using external measures in the magnet environment further compounded by age or disease. As several factors can affect the CVR value determined by ΔCBF/Δend-tidal CO_2_ [34], the usefulness of capnometric measures in the magnet environment are still actively being researched, but have not explained BOLD outcome variability in aged or diseased individuals performing BH tasks. Other factors affecting BOLD outcome during BH is inspirational volume prior to an end-inspirational BH. While chest expansion is a good surrogate to monitor consciously varied inspiration depths prior to BH [12], its success in streamlining natural BH performance in real time is not known. Due to multiple physiological variables involved during the BH, additional physical task burden on subjects are unlikely to eliminate variations in outcome, no matter how hard the subject or patient may try [15]. If fMRI methods improve and evolve towards standard clinical applications, experimental designs with minimal subject discomfort or task burden will assure greater success. To date, there are no studies that have successfully decreased chest expansion variability in subjects naturally performing the BH task. From our prior experience with the BH task [1], [18], [19], [34], we optimized the 20 sec time length which could be completed comfortably by a large number of younger and older subjects. In post-study debriefing and time series inspection of the BH data in every subject, we ascertained that all subjects, irrespective of age, completed the BH task. Inspiration depth-induced BOLD variation was not a concern as subjects avoided inhaling excess air than they would normally when they spontaneously breathed. Inspiration depths requiring no extra efforts were perfected by subjects during a practice session prior to scanning. This practice procedure resulted in consistent BH-induced response with no significant differences in the mean activation volume between the younger and older groups (Figure 7). From prior studies, only deliberate efforts of 90–100% chest expansion (resembling a Valsalva maneuver) produce marked differences in BOLD, while BH inspiration depths variations between 40–80% produce very small BOLD differences [12]. Thus in trained subjects, small inspiration depth variations might not significantly affect BH-induced BOLD responses. Despite the younger and older subjects satisfactorily performing the simple 20 sec end-inspirational BH task, the observed loss of linear relationship with task in the older subjects indicated a different BH-related physiological cause than inspiration depth variations.

### Vasomotive component: a major constituent of low frequency resting state BOLD fluctuations

Resting state low frequency BOLD fluctuations and their temporal characteristics have been frequently used as correlates of intrinsic neural activity [36], [37], [38], [39]. However, low frequency BOLD fluctuations in the resting state also represent end-tidal CO_2_ variations [20] and their temporal and frequency spectral amplitudes have a high voxel-wise correlation with BH-induced BOLD changes [18], [19], [21], [40]. As BOLD signals are a convolution of neural and vascular variables and BOLD responses in the task-related and resting state correlate with each other [17], low frequency BOLD fluctuations cannot be exclusively interpreted as neural or vascular markers but might represent both [41]. The low frequency BOLD is bound to the vasomotive component related to respiration in spontaneously breathing human subjects [21], [42]. Our prior studies, along with other independent evidences correlating end-tidal CO_2_ fluctuations with low frequency BOLD fluctuations have established RSFA as a strong CVR correlate, available from a simple resting state fMRI scan of spontaneously breathing humans with their eyes closed [18], [19], [20]. As decreased resting- state functional connectivity occurs in eyes closed states compared to eyes open [43], [44], state variations in neural activity are minimal during estimation of RSFA in the current ‘eyes closed’ design and ideal for CVR estimates. Low frequency BOLD fluctuations, however, can vary during mind-wandering which is difficult to control. But the overwhelming majority (almost 80–90%) of the low frequency spectral amplitude in spontaneously breathing humans can be attributed to vasomotive factors [42]. Even a subtle respiratory state change such as ‘cued breathing’ during the resting state can tremendously reduce the low frequency vasomotive component by more than 50% [42]. Hence subtle cognitive changes as associated with closed eyes, open eyes or mind-wandering that might alter coherence in neuronal circuits negligibly impact BOLD fluctuation amplitudes compared to vasomotive changes. It is for this reason vasomotive signals (termed as noise) are generally removed to improve resting state functional connectivity analyses of brain functional networks [42]. In other words, the resting state vasomotive component that RSFA represents can be imagined as the maximal response to randomized event-related BH trials of varying short durations (eg., 1, 2, 3 or 4 seconds) depending upon the time-dependent respiratory variability (RVT) within the resting and spontaneously breathing subject.

### Differences between RSFA and BH

RSFA can be considered to be independent of task performance and behavioral variability unlike BH. Our results indicated that the linear relationship between RSFA and task was intact in the younger and older subjects (Figure 3). CVR variability represented by RSFA increased in the older subjects unlike BH. Thus these two variables behave quite differently. A comparative analysis of the subject-wise relationships between BH and RSFA showed significant to near significant linear relationships in the younger subjects within the motor and cognitive task activated areas (Figure 6A and 6B) whereas these relationships were much in the older group (Figure 6C and 6D). Because younger and older subjects performed the motor and BH tasks consistently, the results from Figures 7C and 7D indicated that different subjects within the older group, with quite variable CVRs, might lead to closely clustered BOLD responses during BH that might attenuate the accuracy of CVR estimation in older individuals. These results together indicate that BH might not accurately measure CVR in older individuals, and to a lesser extent, within younger individuals (Figure 6B). Overall, the primary reason for the absence of the linear relationship between BH-motor and BH-cognitive task responses in the older subjects appears, on the basis of our results, to be subject-specific performance and physiological outcome to BH and might not reflect age-related CVR changes *per se* within brain tissue. A notable result was an increased dispersion in RSFA in the motor areas compared to cognitive areas (that included the visual cortex) in older subjects (Figure 3B). This increased dispersion could not be attributed to CSF pulsations as there was also a proportional change in BOLD response to the motor task in these subjects. In an earlier analysis [19], we observed that low pass filtering of the resting state BOLD fluctuations (to remove CSF pulsations) still preserved 85–100% of RSFA's amplitude across the range of gray matter and white matter voxels. This indicated that CSF pulsations do not contribute significantly to RSFA within non-ventricular brain regions and for the same reason, RSFA was considered as a CVR marker without further signal processing such as filtering [19]. While further studies are required to attribute such a difference to selective age-related tissue atrophy in the motor areas, with the present results, one can only speculate to an age-related regional cerebrovascular difference of an unknown origin within motor cortical areas compared to visual cortical areas.

Preprocessing steps such as global mean-subtraction may influence RSFA. In a recent study of young adult humans, global mean-subtracted signals showed a low subject-level correlation between RSFA and task-evoked BOLD amplitude in the visual cortex [29], whereas correlation between coherence of the resting state fMRI signals and task-evoked BOLD amplitude at the voxel- or subject-levels were not affected by global mean removal [29], [45]. However, global mean-removal is unnecessary when estimating the CVR component represented by RSFA [18], [19].

### Normalization of task-evoked BOLD response and RSFA

Hemodynamic scaling or normalization of BOLD responses can account for vascular-related changes within the brain and can correct for vascular differences among inhomogeneous population. Scaling or normalization can be performed in different ways. The first is by dividing the task-induced response by the CVR response on a voxel wise manner [21], [46]. A second approach is to divide the average task-induced response by the average CVR response from the subject-level response [16]. A third approach is to consider the CVR response as a covariate and remove it from the task-induced response [16]. However, a linear relationship between CVR and task response is a prerequisite to estimate normalized or scaled fMRI responses using CVR as a covariate [16]. The present results using a large age range indicate that BH may not satisfy this prerequisite as the linear relationship between BH versus task was disrupted. Hence, the unconstrained RSFA, with an intact linear relationship with tasks, can be used for within-subject normalizations [18] or between-subject normalizations [16] without any age-related or methodological constraints. Furthermore, in choosing a practical normalization variable, RSFA performs better than BH or CO_2_ breathing for several reasons. Generally, vasomotive-related hypercapnic challenges whether BH or CO_2_ breathing are not completely free of neuronal activity components. RSFA also contains neuronal activity components but only ‘spontaneous’ as opposed to ‘evoked’ in the other two cases. For example, BH evokes air hunger leading to excitation of respiratory-related neurons and hypercapnia with CO_2_ ranging from 2% to 5% typically evoke neuronal activity within the brain [19]. Hence, the neuronal components in the BH and CO_2_ challenges are relatively stronger (being evoked neuronal activity) than the spontaneous neuronal components present within RSFA.

### Conclusions

Subject-related differences in physiological changes during BH can profoundly affect CVR determination in large age ranges. The unconstrained CVR marker such as RSFA available from the resting state fMRI signal is suggested as a better alternative to BH. The unconstrained assessment of subtle CVR differences in the higher and lower order brain areas were sensitive enough to detect regional vascular differences and well-suited for fMRI designs that include comparisons between healthy individuals, older individuals, patient- and special-populations [47].

## Supporting Information

Figure S1 Relationship between the subject-average fractional task-induced BOLD signal change (%) versus BH. **A,C.** during motor (fingertap) task and **B,D.** during cognitive (DSST) tasks. **A, B.** indicate older subjects and **C, D.** indicate younger subjects. A significant linear correlation was observed between motor task versus RSFA (r = 0.82; p<0.05) and cognitive task versus RSFA (r = 0.60; p<0.05) in the pooled population of younger and older subjects (n = 22). However, no significant correlation was observed between the motor task versus BH (r = 0.01) and the cognitive task versus BH (r = 0.13) in the pooled population of younger and older subjects.
(TIF)

Figure S2 Relationship between the subject-average fractional BH-induced BOLD signal change (%) versus RSFA. **A.** in the motor (fingertap) task and **B.** in the cognitive (DSST) task activated areas pooled from both younger and older subjects. No linear correlation was observed between BH versus RSFA in the motor areas (r = 0.6) and was greatly reduced in the cognitive task activated areas (r = 0.20).
(TIF)
