# Supplementary figures and images for: Assessment of Unconstrained Cerebrovascular Reactivity Marker for Large Age-Range fMRI Studies
Source: PLoS One. 2014 Feb 13;9(2):e88751. doi: 10.1371/journal.pone.0088751 (PMC3923811; doi:10.1371/journal.pone.0088751)

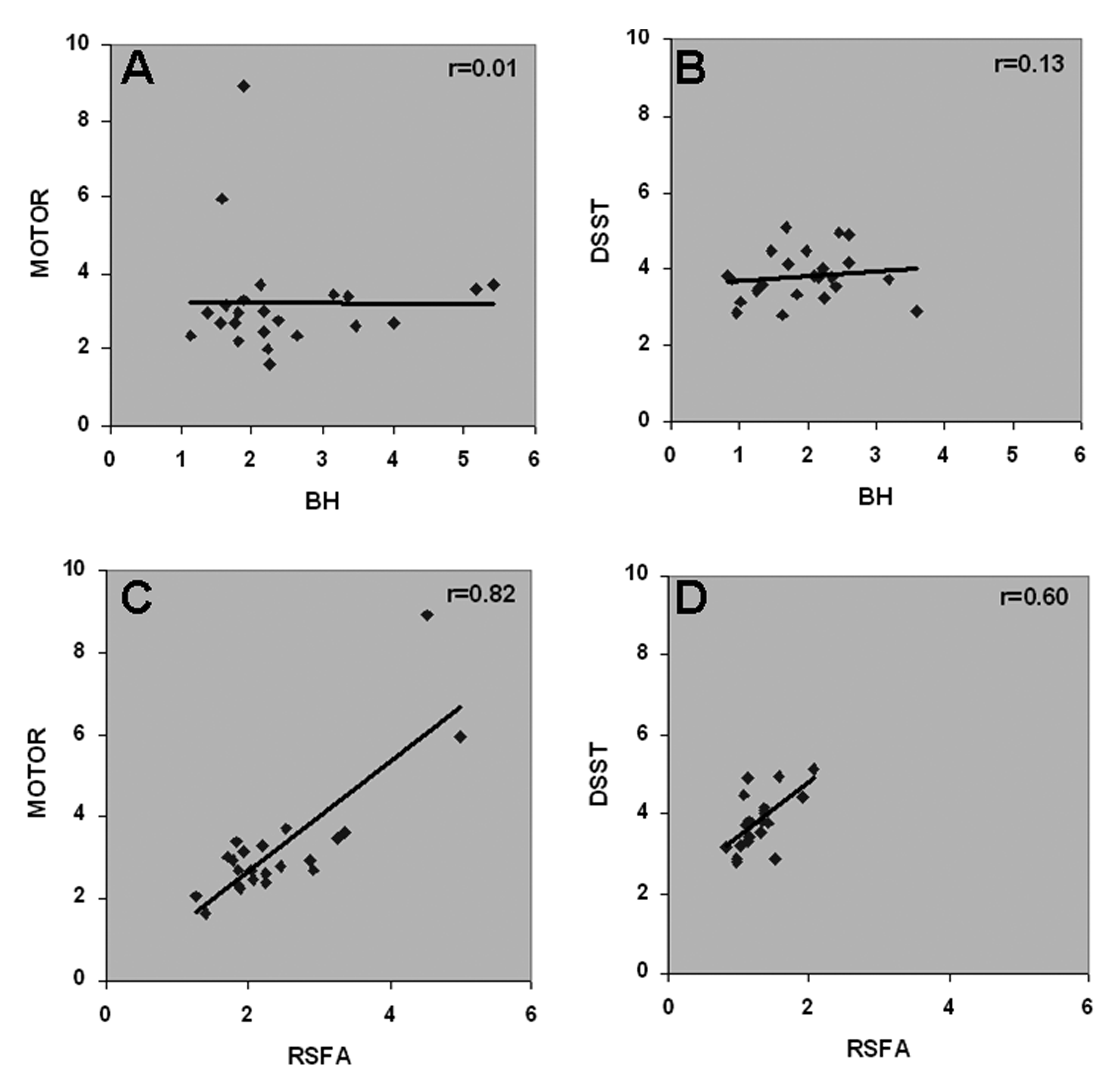

Supplement: Figure S1 — Relationship between the subject-average fractional task-induced BOLD signal change (%) versus BH. A,C. during motor (fingertap) task and B,D. during cognitive (DSST) tasks. A, B. indicate older subjects and C, D. indicate younger subjects. A significant linear correlation was observed between motor task versus RSFA (r = 0.82; p<0.05) and cognitive task versus RSFA (r = 0.60; p<0.05) in the pooled population of younger and older subjects (n = 22). However, no significant correlation was observed between the motor task versus BH (r = 0.01) and the cognitive task versus BH (r = 0.13) in the pooled population of younger and older subjects. (TIF) [file pone.0088751.s001.tif]

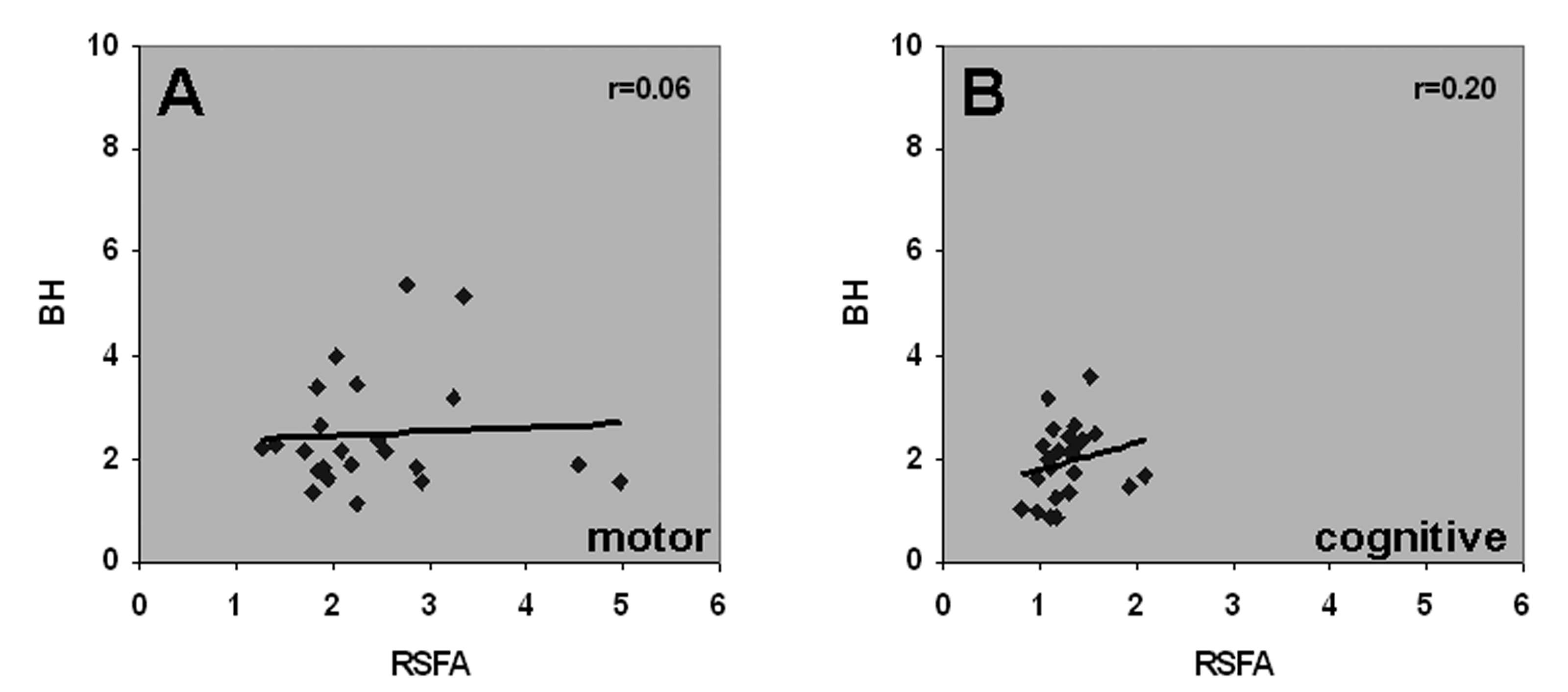

Supplement: Figure S2 — Relationship between the subject-average fractional BH-induced BOLD signal change (%) versus RSFA. A. in the motor (fingertap) task and B. in the cognitive (DSST) task activated areas pooled from both younger and older subjects. No linear correlation was observed between BH versus RSFA in the motor areas (r = 0.6) and was greatly reduced in the cognitive task activated areas (r = 0.20). (TIF) [file pone.0088751.s002.tif]
